# Assessment of risk of intussusception after pilot rollout of rotavirus vaccine in the Indian public health system

**DOI:** 10.1016/j.vaccine.2020.05.093

**Published:** 2020-07-14

**Authors:** Early Rollout of ROTAVAC®India Network, Nita Bhandari, Kalpana Antony, Vinohar Balraj, Temsunaro Rongsen-Chandola, Tivendra Kumar, Bireshwar Sinha, Nidhi Goyal, Rajesh Guleri, Ashish Bavdekar, Sanjay Juvekar, Girish Dayma, Vaijayanti Patwardhan, Archana Patil, Gagandeep Kang, Venkata Raghava Mohan, Rajan Srinivasan, Sridevi A. Naaraayan, Samarasimha Reddy, Maharaj Kishan Bhan, Tataji Surender Rao, Umesh Parashar, Jaya Prakash Muliyil, Jaqueline Tate, Nicholas J. Andrews, Prasanna Samuel, Santhosh Kumar Ganesan, Sunita Taneja, Tarun Shankar Choudhary, Veereshwar Bhatnagar, Arun Kumar Gupta, Madhulika Kabra

**Affiliations:** a Centre for Health Research and Development, Society for Applied Studies, New Delhi, India; b PATH, India; c District Health Office, Ministry of Health, Kangra, Himachal Pradesh, India; d KEM Hospital Research Centre, Pune, Maharashtra, India; e State Family Welfare Bureau, Public Health Services, Government of Maharashtra, Maharashtra, India; f Christian Medical College, Vellore, Tamil Nadu, India; g Department of Pediatrics, Institute of Child Health, Chennai, India; h Department of Community Medicine, Santhiram Medical College and General Hospital, Andhra Pradesh, Hyderabad, India; i Indian Institute of Technology, New Delhi, India; j Viral Gastroenteritis Branch, Centers for Disease Control and Prevention, Atlanta, USA; k Public Health England, London, United Kingdom; l All India Institute of Medical Sciences, New Delhi, India

**Keywords:** Rotavirus vaccine, Intussusception, Vaccine safety

## Abstract

**Background:**

Pre-licensure trials of ROTAVAC® were not adequately powered to assess risk of intussusception, a rare adverse event associated with other rotavirus vaccines in some settings. We examined the risk of intussusception after ROTAVAC® vaccination among Indian infants during pilot rollout of the vaccine in the public health system in three states - Himachal Pradesh, Maharashtra and Tamil Nadu.

**Methods:**

Passive surveillance for intussusception was set up in 35 sentinel health facilities covering 26.3 million population in the three states under monitoring of an Interministerial-Interagency Steering Committee. Clinical and immunization data were collected by independent teams. An expert committee blinded to vaccination status, classified intussusception cases using Brighton criteria. The self-controlled case-series method was used to estimate risk of intussusception (Brighton Level 1) after ROTAVAC® vaccination.

**Results:**

151 intussusception cases were included in the analysis. The relative incidence (incidence during the risk period compared to the control period) 1–21 days after doses 1 and 2 combined was 1.56 (95% CI, 0.0–5.28) and that for three doses combined was 1.88 (95% CI, 0.76–4.30). Attributable risk 1–21 days after doses 1 and 2 combined was 0.11 (95% CI, 0.0–0.25) and that for 3 doses combined was 0.42 (95% CI, 0.0–0.70) per 100,000 doses.

**Conclusions:**

No increased risk of intussusception within 21 days of receipt of the first two doses combined or all 3 doses combined of ROTAVAC® was detected.

## Highlights

• Prelicensure trials of ROTAVAC® not powered to assess risk of intussusception.
• During ROTAVAC® rollout we assessed risk of intussusception in infants in 3 states.
• No increased risk of intussusception within 21 days of 1st and 2nd dose.
• No increased risk of intussusception within 21 days of any single dose or all 3 doses.

## 1 Introduction

Rotavirus is the commonest cause of severe dehydrating diarrhea worldwide, causing 215,000 under-five deaths annually and India accounts for ∼20% of these deaths [1]. An Indian vaccine (ROTAVAC®) developed from 116E strain found protective against severe rotavirus gastroenteritis [2] was licensed for production and use in India in January 2014.

In 1999, a rotavirus vaccine (Rotashield, Wyeth) was withdrawn from United States due to an excess risk of intussusception i.e. ∼1 to 2 cases per 10,000 recipients [3], [4]. For RotaTeq® and Rotarix™ vaccines, studies have documented a risk of ∼1 to 6 excess cases of intussusception per 100,000 vaccinated infants in USA, Australia, England, Mexico and Brazil [5], [6], [7], [8], while in Sub-Saharan and South Africa no increased risk of intussusception is associated with Rotarix [9], [10]. Large benefits and low risk of intussusception led to global policy in support of routine use of rotavirus vaccines in national immunization programs [11]. However, the World Health Organization recommended monitoring for intussusception risk with introduction of newer rotavirus vaccines [11].

ROTAVAC® phase III clinical trial in India with 6799 infants found no increased risk of intussusception but was not powered to detect low intussusception risk [2]. In 2014, prior to introduction of ROTAVAC® nationally in India, the Indian National Technical Advisory Group on Immunization recommended monitoring for rare side effects like intussusception during a phased rollout of ROTAVAC® in the public health system in three states - Himachal Pradesh in North, Maharashtra in West and Tamil Nadu in South India. Using the self-controlled case series (SCCS) methodology, we estimated the risk of intussusception (Brighton Diagnostic Criteria Level I [12]) within 21 days after the first two doses of ROTAVAC® in the three sites.

## 2 Material and methods

### 2.1 Study management and coordination

The pilot rollout of ROTAVAC® along with surveillance for intussusception was conducted under the oversight of an interministerial-interagency steering committee jointly chaired by Secretary, Department of Biotechnology; Secretary, Department of Health Research, Government of India and Director General of the Indian Council of Medical Research. This committee facilitated support to state and district authorities, reviewed implementation plan and technical decisions, assisted by the Secretariat at Biotechnology-Industry-Research-Assistance-Council and Centre for Health Research and Development, Society for Applied Studies (CHRD-SAS), New Delhi for project coordination. A Project Management Committee with senior scientists and government representatives provided continued technical guidance.

### 2.2 Study sites and population

At each site surveillance was coordinated by study partners; CHRD-SAS, Delhi in Himachal Pradesh, KEM Hospital Research Centre, Pune in Maharashtra and Christian Medical College, Vellore in Tamil Nadu. ROTAVAC® was introduced at the same time surveillance for intussusception started in the respective sites/states.

In Himachal Pradesh, surveillance started in Kangra district in December 2015 and expanded to nine more districts Mandi, Bilaspur, Una, Hamirpur, Chamba, Shimla, Sirmaur, Solan and Kullu (population ∼7 million). In Maharashtra, surveillance started in December 2015 in Junnar and Ambegaon blocks and parts of Shirur and Khed blocks in Pune district (population ∼900,000). In Tamil Nadu, surveillance started in September 2017 in Vellore district and expanded to Chennai, Kanchipuram and Tiruvallur districts covering 47 rural blocks, 24 municipalities and 2 corporations (population ∼18.4 million).

As per the national programme, three doses are recommended at 6, 10 and 14 weeks of age; the first dose could be given up to 1 year of age [13]. Later, ROTAVAC® was marketed as ROTASURE at some study sites by another pharmaceutical and used only in the private sector. ROTAVAC® and ROTASURE were considered as the same vaccine for our monitoring purposes. Vaccine introduction and surveillance was integrated into existing primary health care of the state and local government at each site. As an integral part of primary health care, individual consent for collection of information was not considered necessary. Study teams however obtained necessary approval from respective Institutional Review Boards.

### 2.3 Establishment of passive surveillance

In 2015–16, all three sites determined healthcare utilization patterns for acute conditions in young children in their study populations including local healthcare provider details and referral pathways to higher facilities. This identified potential facilities that served ≥80% under-two children in the study population. Quantitative and qualitative methods were used to interview healthcare staff, key informants and parents. From the information obtained, healthcare facilities were identified and assessed using a questionnaire for availability of paediatricians, paediatric surgeon, ultrasound, experience in managing or referral of acute abdominal conditions such as intussusception in the prior five years. Thirty-five facilities were chosen as sentinel hospitals for passive surveillance of intussusception across the sites; 6 in Tamil Nadu, 11 in Pune and 18 in Himachal Pradesh. In 2018, the sites carried out an exercise to identify changes in referral pathways and newer treatment facilities in the study areas, none were identified or added.

Within each sentinel facility, workflows for pathways of child admission with possible intussusception were drawn-up (emergency/ outpatient/pediatric wards/neonatal or pediatric intensive care). Each sentinel hospital had a clinical team with nodal person(s) monitoring and maintaining logs of possible cases by screening hospital-based registers including those in the radiology department and operation theatre. For all possible cases identified, hospital records and digital or scanned copies of images were obtained. A clinical record summary was filled documenting date of birth, onset, clinical details, investigations, diagnosis, treatment and outcomes. Each record was rechecked by a medical officer of the clinical team. There was no direct contact of the clinical team with the infants or families. Another independent team collected immunization details from immunization cards or records available at primary health center, sub-center or hospitals. Periodic audits at each site by an independent expert looked for possible cases missed and data quality. Proof of date of birth, rotavirus immunization date, evidence of Brighton level 1 were mandatory and source document copy was obtained.

### 2.4 Data management

Data collection at each site started with intussusception surveillance and ROTAVAC® rollout in December 2015, with one site starting later in September 2017, and ended in July 2019 with one site ending earlier in February 2019. Sharepoint created for uploading the clinical record summary, supporting documents (digital or scanned images) was used by the Coordination Unit through role-based access control for checking completeness of mandatory information and quality.

### 2.5 Diagnosis of intussusception - rare side effects case adjudication committee (RSEAC)

All cases were made available to the RSEAC after blinding for immunization history. The RSEAC had an independent paediatric surgeon; paediatrician and radiologists who classified intussusception cases using Brighton Diagnostic Criteria [12] based on available evidence and provided a signed report.

### 2.6 Monitoring vaccine use at study sites and vaccine coverage survey

The sites collected births and total doses of ROTAVAC® used in study areas from the government every month.

Prior to end of surveillance, a probability-based 30-cluster vaccine coverage survey was carried out at each study site [14]. In each cluster, 16 children aged 5–23 months, age-eligible to have completed 3-doses of rotavirus vaccine in the current or previous year were selected by computer-generated random numbers from the list of all children available from government sources. Information on the types and source (government or private) of the rotavirus vaccination were documented. This was used to estimate rotavirus vaccine doses administered by private sources which added to doses from government sources provided total corrected ROTAVAC® vaccine doses used in the study areas.

### 2.7 Eligibility for primary and secondary analysis

Infants from the study area with Brighton Level 1 (BL1) intussusception (only first event) and adequate documentation were eligible. The date of first imaging for diagnosis (ultrasound/ CT/fluoroscopy), prior to intervention was the date of onset of intussusception. If only X-ray was available prior to intervention, the date of first X-ray reporting an intestinal obstruction was considered as date of onset, but proof of intussusception was required by surgery or imaging at reduction. The vaccine (exposure) of primary interest was ROTAVAC®. Unvaccinated cases were included for age adjustments in both primary and secondary analyses. Brighton level 2 (BL2) cases were included in the sensitivity analysis. Cases receiving other rotavirus vaccines (Rotateq/Rotarix/ Rotasiil) alone or in combination with ROTAVAC® were excluded from analysis.

### 2.8 Observation, risk and control periods

The observation period for each case was 28–365 days of age. Each child could contribute a maximum of 337 days but if the start of the follow-up was after 28 days of age or end of follow-up was before 365 days of age, the child would contribute fewer person days. The observation period was divided into risk and control periods following vaccination; risk period was 1–21 days post-receipt of each of the three doses of ROTAVAC® vaccine, control period was 22–365 days of age, excluding the 1–21 days after each dose of vaccination. The primary risk period of 1–21 days post-vaccination was based on the timing of intestinal replication of the vaccine virus and peak period of risk observed with the other available rotavirus vaccines [6], [9], [10], [15], [16]. We also examined the risk of intussusception separately for periods 1–7 days and 8–21 days post-vaccination compared to control period. For unvaccinated cases, follow-up started from age at day 28 (or age at start of surveillance) and ended at 365 days (or age at end of surveillance) and all person-time was non-risk or control period.

### 2.9 Sample size

Sample size was calculated accounting for age-vaccine exposure collinearity using a simulation approach based on the age distribution of primary vaccinations and intussusception in the study area. Eighty cases who had received one or more doses of ROTAVAC® were sufficient to detect a relative incidence (RI) of 4 across doses 1 and 2 (1–21 period) with 80% power and 2-sided significance level of 5%, assuming a three-dose vaccine coverage of at least 50%. For each dose this number was sufficient to detect an RI of ∼3.5 for third dose, 4.5 for second dose and 6 for first dose.

### 2.10 Statistical analysis

We used SCCS method to estimate the risk of intussusception following rotavirus vaccine during risk periods compared to control periods [17] similar to several countries [6], [8], [9], [10]. Since rotavirus vaccination is contraindicated after an episode of intussusception, the event dependent version of the SCCS was used [17], [18], [19]. Age was adjusted by dividing the observation period of each child into 4-week age-intervals; it allowed best capture of the shape of age-distribution of intussusception and avoided zero counts in age groups. Data from unvaccinated cases helped define the age-effects which can be difficult to distinguish from vaccine effects if only using vaccinated cases due to the fairly narrow range of ages at vaccination. In the primary SCCS analysis we estimated the relative incidence (incidence during the risk period compared to control period) of intussusception within 1–21 days after doses 1 and 2 combined, after adjusting for the risk in the post-dose 3 period. In our secondary analyses, we estimated the RI of intussusception in risk periods 1–7, 8–21 and 1–21 days post each dose (first, second and third) of vaccination and all doses combined. Stata version 16, TX, USA [20] was used, 95% confidence interval (CI) were estimated by bootstrapping with 1,000 iterations. Sensitivity analysis was conducted to test robustness of the results from main analyses after including eligible BL2 cases.

Attributable risk of intussusception per 100,000 ROTAVAC® doses was calculated using the formula [100,000 * ((no. of cases in the risk window) * ((RI − 1)/RI) ÷ (no. of total vaccine doses))] for doses 1 and 2 and for all three doses combined.

## 3 Results

**Fig. 1:**
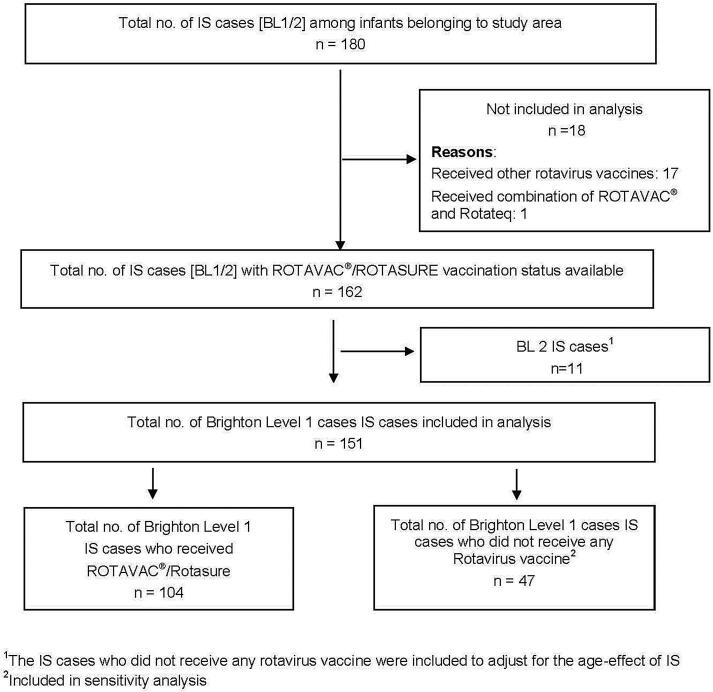
Flowchart showing the intussusception cases and those included or excluded from the analysis.

A total of 180 infants were reviewed by RSEAC and classified as BL1 or BL2. Of these, 18 had received other rotavirus vaccines (Rotarix, Rotateq, Rotasiil) alone or in combination with ROTAVAC® and were not included in the analysis. Of the 162 remaining cases, 151 were BL1 and 11 were BL2. Among the 151 BL1 cases included in analysis, 104 received ROTAVAC® and 47 were unvaccinated (Fig. 1).

The median (IQR) age of the BL1 cases was 222 days (178–271), two-thirds were male, ∼80% had ileocolic intussusception and 40% (61/151) of BL1 cases had surgical reduction. One in-hospital death was reported in a 286-day-old with BL1, ileocolic type intussusception with pyoperitoneum and sepsis who had not received any rotavirus vaccine. The total estimated doses of ROTAVAC® used in the study areas during the study period was 493,687, 477,372 and 464,167 for first, second and third doses respectively. The average immunization coverage across the three sites for the three doses of ROTAVAC® was 91%, 87% and 81%, respectively.

**Fig. 2:**
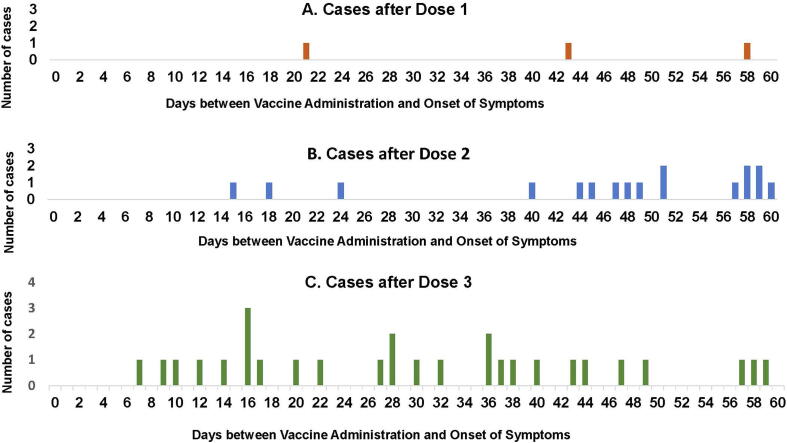
Cases of Intussusception after the three doses of ROTAVAC® vaccine.

**Fig. 3:**
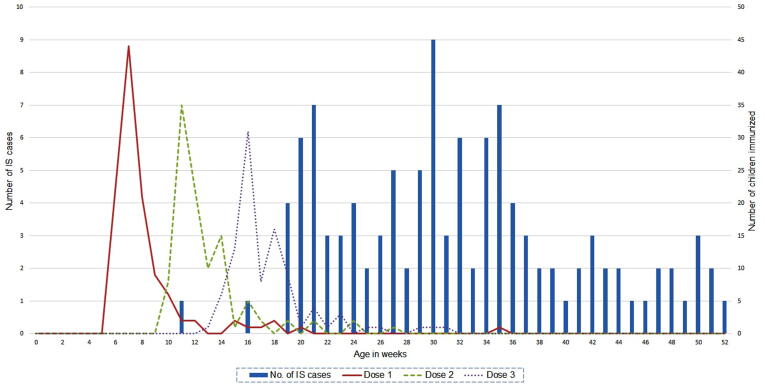
Age at immunization and onset of intussusception in infants.

Of 104 BL1 cases who received ROTAVAC®, 1, 2 and 10 cases had an onset within 1–21 days of receipt of doses 1, 2 and 3 respectively. In the control periods, between day 22 of dose 1 and dose 2 and between day 22 of dose 2 and dose 3, there were 7 cases each; while 77 cases had an onset between day 22 of dose 3 and 365 days of age. No clustering of cases was observed after any vaccine dose (Fig. 2). The median age at receipt of ROTAVAC® doses 1, 2 and 3 in 104 BL1 cases was 50, 84 and 116 days respectively. More than 75% of the intussusception cases were beyond the median age of the third dose (Table 1, Fig. 3).

**Table 1:** Description of the Intussusception Cases with Brighton Level 1 or 2.

	BL 1 cases N = 151 n (%)^a^	All (BL1 & BL2 cases) N = 162 n (%)^a^
**Age at onset (in days):** Mean (SD) Median (IQR)	227.5 (65.1) 222.0 (178.0–271.0)	226.3 (64.6) 220.5 (178.0–269.0)
**Sex**: Female	51 (33.7)	55 (33.9)
**Intestinal location of intussusception** Ileocolic Colocolic Ileoileal Others^b^ Unconfirmed location	123 (81.5) 4 (2.7) 6 (3.8) 10 (6.6) 8 (5.3)	127 (78.4) 4 (2.5) 8 (4.9) 11 (6.8) 12 (7.4)
**Rotavirus vaccine received**^c^ Not received ROTAVAC®	47 (31.1) 104 (68.9)	49 (30.3) 113 (69.7)
**ROTAVAC**® **vaccination doses** Dose 1 Dose 2 Dose 3	104 (100.0) 96 (92.3) 92 (88.5)	113 (100.0) 104 (92.0) 100 (88.5)
**Age (in days) at vaccination, Median (IQR)** Dose 1 Dose 2 Dose 3	50.0 (46.0–57.5) 84.0 (77.0–96.0) 116.0 (110.0–129.0)	50.0 (46.0–57.0) 83.0 (77.0–96.0) 115.0 (110.0–129.0)
Reduction by Surgical methods Surgery with bowel loss or stoma	61 (40.4) 14 (9.3)	61 (37.6) 14 (8.6)
**Outcome** Discharged Death	150 (99.3) 1 (0.7)	160 (99.4) 1 (0.6)

BL1 = Brighton Level 1; BL2 = Brighton Level 2.

a Unless otherwise specified.

b Includes - ileo-colocolic, ileo-ileocolic, caecocolic, ileocaecal, ileocaecocolic and jejunduodenal.

c 6/104 received ROTASURE; 8/113 received ROTASURE.

Primary analysis showed no increased risk of intussusception during days 1–21 after receipt of dose 1 and 2 of ROTAVAC® vaccine compared to the control period (RI 1.56, 95% CI, 0.0–5.28). Secondary analysis showed no significant increase in risk of IS during days 1–21 after dose 1, 2 and 3 individually or all doses combined, compared to the control period (Table 2). There was no increase in risk of intussusception for 1–7 days or 8–21 days after doses 1, 2 and 3 individually or all doses combined, compared to the control period. The results of the sensitivity analyses were similar (Table 2).

**Table 2:** Case Counts and Relative Incidence of Confirmed Intussusception Cases after ROTAVAC® Vaccination in Indian Infants.^a^

Doses of Rotavirus vaccines	Risk period	No. of BL1 cases	RI (95% CI) in the risk period^b^
**Primary Analysis**			
Combined dose (1 + 2)^c^	1–21	3	1.56 (0.0–5.28)

**Secondary Analysis**			
Dose-1	1–21	1	1.75 (0.0–7.80)
Dose-2	1–21	2	1.51 (0.0–5.58)
Dose-3	1–21	10	2.02 (0.74–4.88)
Combined all doses	1–21	13	1.88 (0.76–4.30)
Combined Dose (1 + 2)	1–7	0	–
Combined Dose (1 + 2)	8–21	3	2.00 (0.0–6.44)
Dose-3	1–7	1	0.71 (0.0–3.25)
Dose-3	8–21	9	2.36 (0.73–5.23)
Dose-1	1–7	0	–
Dose-1	8–21	1	2.26 (0.0–8.87)
Dose-2	1–7	0	–
Dose-2	8–21	2	1.92 (0.0–6.79)
Dose-3	1–7	1	0.71 (0.0–3.23)
Dose-3	8–21	9	2.36 (0.74–5.20)
Combined all doses	1–7	1	0.51 (0.0–2.38)
Combined all doses	8–21	12	2.16 (0.89–4.46)

**Sensitivity analysis** (including BL2 cases)			
Combined Dose-1 + 2^c^	1–21	3	1.20 (0.0–4.18)
Dose-1	1–21	1	0.96 (0.0–5.25)
Dose-2	1–21	2	1.30 (0.0–4.91)
Dose-3	1–21	10	1.65 (0.65–3.84)
Combined all doses	1–21	13	1.51 (0.58–3.23)

BL1 = Brighton Level 1; BL2 = Brighton Level 2.

a Intussusception cases in the control periods: (a) after 21 days of first dose and before second dose = 7; (b) after 21 days of second dose and before third dose = 7; (c) intussusception cases after 21 days of the third dose till age of 365 days = 77.

b 95% CI generated via bootstrapping with 1000 iterations.

c After adjusting for the risk in the post-dose 3 period.

Attributable fraction and the estimated number of vaccine attributable cases for doses 1 and 2 combined was 36% and 1.08, respectively. For all 3 doses combined it was 47%, and 6.09, respectively. The attributable risk in the 1–21-day period after doses 1 and 2 combined was 0.11 (95% CI, 0.0–0.25) and for all doses combined was 0.42 (0.0–0.70) per 100,000 doses.

## 4 Discussion

Our study showed no increased risk of intussusception associated with any of the three doses of the ROTAVAC® vaccine or combinations of doses within 21 days of vaccination compared to the control period in the study sites.

These findings contrast with earlier studies in some high and upper middle income countries where rotavirus vaccination was associated with intussusception [6], [7], [8], [21] but similar to reports from Sub-Saharan [9] and South Africa [10] where risk of intussusception during 1–21 days of first or second dose of Rotarix was not higher than the background risk i.e. risk in the control period.

Some factors may explain differences in the risk between high versus low income settings. First, the recommended ROTAVAC® schedule in India at 6, 10 and 14 weeks of age is similar to the two-dose Rotarix schedule (6 and 10 weeks) in Sub-Saharan Africa and South Africa, whereas in high and middle income settings the vaccine is administered later at ∼2 and 4 months. Incidence of intussusception in the first two months of life is low and administering vaccine at this age may hence pose less risk [10], [16]. Second, it is postulated that intussusception may be related to intestinal viral replication [9], [16]. In India and other lower-middle income countries the efficacy of this oral vaccine is lower [2], [22] compared to the high income countries [23] suggesting lower intestinal viral replication and hence lower risk of intussusception. Third, rotavirus vaccine and oral polio vaccine are given simultaneously in the Indian schedule which has demonstrated a reduction in the immunogenicity of the rotavirus vaccine [24] and potentially lower vaccine virus replication.

Among our strengths, this is the first organized effort in India to generate safety data on intussusception after rotavirus vaccine introduction in the public health system using passive surveillance. This study was adequately powered to exclude a RI of 4 or more for a vaccine coverage of 50%. We had 104 cases and a vaccine coverage of >80% against a calculated sample size of 80 and coverage of 50%. Supervision by the steering and project management committees ensured protocol was followed, all cases analysed had adequate and verifiable documentation needed for Brighton classification and inclusion/exclusion in the analysis. Clinical data teams were blinded to immunization status which was obtained by a separate team. The RSEAC that reviewed each case was independent and blinded to immunization status. Hence, information and misclassification bias were avoided as best as was possible.

Among limitations, any delay in care-seeking may result to intussusception-related deaths before reaching health facility which are not captured. However, this possibility being independent of vaccination is not a concern for biased results. Documented date of first imaging was apriori considered as the date of onset of intussusception because of lack of uniformity in clinical history recording and investigation practices at different health facilities. In a three-tier heath care system, a child could reach a sentinel facility with imaging done ahead of admission for intervention. Our mean (SD) difference between date of onset (first ultrasound or other imaging) and admission was 0.2 (0.7) days. There were 15/104 BL1 cases with date of admission earlier than the date of onset (admission before investigations) but none shifted from the control to risk window (1–21 post-vaccination) or vice-versa had we considered date of admission as the date of onset of IS. We acknowledge that power of the study is limited to exclude a low or very-low risk, since we were looking at RI of 4 in 1–21 days of doses 1 and 2 combined.

## 5 Conclusions

Experience from the pilot rollout of ROTAVAC® in the Indian public health system provides data to support no increased risk of intussusception in infants after any individual dose of the vaccine or in combination, compared to the risk in the control period. Timeliness of vaccine administration is important since risk of intussusception incidence increases with infant-age. The large health benefits and absence of excess risk of intussusception associated with the vaccine is reassuring. Nonetheless, given that intussusception is known to vary by geography and is influenced by local dietary practices [3], more data to support safety of the vaccine will be valuable as it is being rolled-out in other parts of the country.

## Funding

The project was funded by 10.13039/100000865Bill & Melinda Gates Foundation, Seattle, WA [grant number OPP1086457]. The funding agency did not play any role in study design; in the collection, analysis and interpretation of data; in the writing of the report; and in the decision to submit the article for publication.

## CRediT authorship contribution statement

**Nita Bhandari:** Conceptualization, Methodology, Writing - original draft, Writing - review & editing, Supervision, Project administration, Funding acquisition. **Kalpana Antony:** Data curation, Writing - original draft, Writing - review & editing, Project administration. **Vinohar Balraj:** Methodology, Software, Investigation, Data curation, Formal analysis, Writing - original draft, Writing - review & editing, Visualization, Project administration. **Temsunaro Rongsen-Chandola:** Investigation, Supervision, Writing - review & editing. **Tivendra Kumar:** Investigation, Writing - review & editing. **Bireshwar Sinha:** Investigation, Data curation, Formal analysis, Writing - original draft, Writing - review & editing, Visualization, Project administration. **Nidhi Goyal:** Investigation, Writing - review & editing. **Rajesh Guleri:** Writing - review & editing, Supervision. **Ashish Bavdekar:** Investigation, Supervision, Writing - review & editing. **Sanjay Juvekar:** Investigation, Supervision, Writing - review & editing. **Girish Dayma:** Investigation, Data curation, Formal analysis, Writing - original draft, Writing - review & editing. **Vaijayanti Patwardhan:** Investigation, Writing - review & editing. **Archana Patil:** Writing - review & editing. **Gagandeep Kang:** Investigation, Supervision, Writing - review & editing. **Venkata Raghava Mohan:** Investigation, Writing - review & editing. **Rajan Srinivasan:** Investigation, Writing - review & editing. **Sridevi A. Naaraayan:** Investigation, Writing - review & editing. **Samarasimha Reddy:** Investigation, Writing - review & editing. **Maharaj Kishan Bhan:** Conceptualization, Methodology, Resources, Supervision, Writing - review & editing. **Tataji Surender Rao:** Supervision, Writing - review & editing. **Umesh Parashar:** Methodology, Resources, Formal analysis, Visualization, Writing - original draft, Writing - review & editing. **Jaya Prakash Muliyil:** Supervision, Writing - review & editing, Formal analysis. **Jaqueline Tate:** Methodology, Resources, Formal analysis, Visualization, Writing - original draft, Writing - review & editing. **Nicholas J. Andrews:** Methodology, Resources, Software, Validation, Formal analysis, Visualization, Writing - original draft, Writing - review & editing. **Prasanna Samuel:** Software, Validation, Data curation, Formal analysis, Visualization, Writing - review & editing. **Santhosh Kumar Ganesan:** Software, Validation, Data curation, Formal analysis, Visualization, Writing - review & editing. **Sunita Taneja:** Formal analysis, Visualization, Writing - review & editing. **Tarun Shankar Choudhary:** Validation, Formal analysis, Visualization. **Veereshwar Bhatnagar:** Investigation, Writing - review & editing. **Arun Kumar Gupta:** Investigation, Writing - review & editing. **Madhulika Kabra:** Investigation, Writing - review & editing.

## Declaration of Competing Interest

The authors declare that they have no known competing financial interests or personal relationships that could have appeared to influence the work reported in this paper.
