# Supplementary material for: Assessment of risk of intussusception after pilot rollout of rotavirus vaccine in the Indian public health system
Source: Vaccine. 2020 Jul 14;38(33):5241–8. doi: 10.1016/j.vaccine.2020.05.093 (PMC7347004; doi:10.1016/j.vaccine.2020.05.093)
Supplement: Supplementary data 1 [file mmc1.docx]

**EARLY ROLLOUT OF ROTAVAC^®^ INDIA NETWORK^1^**

**List of Collaborators**

**Coordination Unit**: Nita Bhandari^a^, Kalpana Antony^b^; Vinohar Balraj^a^

**Study Site - Himachal Pradesh, India**: Temsunaro Rongsen-Chandola^a^, Tivendra Kumar^a^, Bireshwar Sinha^a^, Nidhi Goyal^a^, Rajesh Guleri^c^

**Study Site - Pune, Maharashtra, India**: Ashish Bavdekar^d^, Sanjay Juvekar^d^, Girish Dayma^d^, Vaijayanti Patwardhan^d^, Archana Patil^e^

**Study Site - Tamil Nadu, India**: Gagandeep Kang^f^, Venkata Raghava Mohan^f^, Rajan Srinivasan^f^, Sridevi A Naaraayan^g^, Samarasimha Reddy^h^

**Project Management Committee**: Maharaj Kishan Bhan^i^, Tataji Surender Rao^i^, Umesh Parashar^j^, Jaya Prakash Muliyil^f^, Jaqueline Tate^j^

**Analysis Group**: Nicholas J Andrews^k^, Prasanna Samuel^f^, Santhosh Kumar Ganesan^f^, Sunita Taneja^a^, Tarun Shankar Choudhary^a^

**Rare Side Effects Case Adjudication Committee**: Veereshwar Bhatnagar^l^, Arun Kumar Gupta^l^, Madhulika Kabra^l^

**Writing Committee**: Bireshwar Sinha^a^, Umesh D Parashar^j^, Jacqueline Tate^j^, Nita Bhandari^a^, Kalpana Antony^b^, Nicholas J Andrews^k^, Vinohar Balraj^a^

^a^Centre for Health Research and Development, Society for Applied Studies, New Delhi, India

^b^PATH, India

^c^District Health Office, Ministry of Health, Kangra, Himachal Pradesh, India

^d^KEM Hospital Research Centre, Pune, Maharashtra, India

^e^State Family Welfare Bureau, Public Health Services, Government of Maharashtra, Maharashtra, India

^f^Christian Medical College, Vellore, Tamil Nadu, India

^g^Department of Pediatrics, Institute of Child Health, Chennai, India

^h^Department of Community Medicine, Santhiram Medical College and General Hospital, Andhra Pradesh, Hyderabad, India

^i^Indian Institute of Technology, New Delhi, India

^j^Viral Gastroenteritis Branch, Centers for Disease Control and Prevention, Atlanta, USA

^k^Public Health England, London, United Kingdom

^l^All India Institute of Medical Sciences, New Delhi, India
